# A Mobile App for Promoting Breastfeeding-Friendly Communities in Hong Kong: Design and Development Study

**DOI:** 10.2196/64191

**Published:** 2025-01-10

**Authors:** Heidi Sze Lok Fan, Emily Tsz Yan Leung, Ka Wing Lau, Janet Yuen Ha Wong, Edmond Pui Hang Choi, Christine Lam, Marie Tarrant, Hextan Yuen Sheung Ngan, Patrick Ip, Chia Chin Lin, Kris Yuet Wan Lok

**Affiliations:** 1 School of Nursing, Faculty of Health and Social Development, The University of British Columbia, Kelowna, BC, Canada; 2 School of Nursing, Li Ka Shing Faculty of Medicine, University of Hong Kong, 5/F, Academic Building, Pokfulam, Hong Kong, China (Hong Kong), 852 39176690; 3 School of Nursing and Health Sciences, Hong Kong Metropolitan University, Hong Kong, China (Hong Kong); 4 Department of Obstetrics and Gynaecology, Queen Elizabeth Hospital, Kowloon, Hong Kong, China (Hong Kong); 5 Department of Obstetrics and Gynaecology, Li Ka Shing Faculty of Medicine, University of Hong Kong, Hong Kong, China (Hong Kong); 6 Li Ka Shing Faculty of Medicine, Department of Paediatrics and Adolescent Medicine, University of Hong Kong, Hong Kong, China (Hong Kong)

**Keywords:** Baby-Friendly Community Initiative, Baby-Friendly Hospital Initiative, breastfeeding, community, stakeholders, mobile app, friendly communities, baby-friendly, well-being, mother, infant, application, mHealth, qualitative, user-friendly, self-management

## Abstract

**Background:**

Breastfeeding is vital for the health and well-being of both mothers and infants, and it is crucial to create supportive environments that promote and maintain breastfeeding practices.

**Objective:**

The objective of this paper was to describe the development of a breastfeeding-friendly app called “bfGPS” (HKU TALIC), which provides comprehensive territory-wide information on breastfeeding facilities in Hong Kong, with the goal of fostering a breastfeeding-friendly community.

**Methods:**

The development of bfGPS can be categorized into three phases, which are (1) planning, prototype development, and preimplementation evaluation; (2) implementation and updates; and (3) usability evaluation. In phase 1, a meeting was held with experts, including maternal and child health researchers, app developers, breastfeeding individuals, and health professionals, to discuss the focus and functionality of the breastfeeding app. A prototype was developed, and breastfeeding facilities in various public venues in Hong Kong were assessed using a structured checklist. For the preimplementation evaluation, 10 focus groups and 19 one-on-one interviews were conducted between May 2019 and October 2020 with staff working in public premises (n=29) and breastfeeding individuals (n=29). For phase 2, bfGPS was published on iOS (Apple Inc) and Android (Google) platforms in September 2020. App updates were launched in September 2021 and May 2022 based on the suggestions provided by the participants in the preimplementation evaluation. For the usability evaluation, semistructured, in-depth, one-to-one interviews were conducted with breastfeeding individuals (n=30) to understand their experiences of using bfGPS. Content analysis was used to analyze the data.

**Results:**

bfGPS is a mobile app that was developed to assist breastfeeding individuals in locating breastfeeding facilities in public venues in Hong Kong. In the preimplementation evaluation, the participants gave comments on the layout and interface of bfGPS, and suggestions were given on incorporating new functions into the app. Based on the suggestions of the participants in the preimplementation evaluation, a few additional functions were added into bfGPS, including allowing the users to rate and upload recent information about breastfeeding facilities and an infant tracker function that encourages users to record infant development. In the usability evaluation, 3 main themes emerged—bfGPS improves the community experience for breastfeeding individuals, facilitates tracking the infant’s growth, and provides suggestions for further development.

**Conclusions:**

The bfGPS app is the first user-friendly tool designed to assist users in locating breastfeeding facilities within the community. It stands as a guide for similar health care app developments, emphasizing the importance of accurate, current data to ensure user adoption and long-term use. The app’s potential lies in the support and reinforcement of breastfeeding practices coupled with self-management strategies.

## Introduction

### Background

Breastfeeding provides numerous benefits to both parents and infants [1]. It is considered one of the most effective health interventions for preventing infant and child mortality [2]. The World Health Organization (WHO) recommends that infants be exclusively breastfed for the first 6 months of life. After 6 months of age, complementary feeding should be provided with continuing breastfeeding up to 2 years of age or beyond [3]. However, breastfeeding individuals face numerous challenges that can lead to early weaning. Less than one-half of infants under 6 months old were exclusively breastfed globally, and only 45% of infants can be breastfed up to 2 years of age [4]. The determinants of breastfeeding continuation are multifactorial, with various demographic [5,6], psychological [7], and social factors [8].

Breastfeeding support is crucial for long-term breastfeeding. Technology-based interventions effectively improve breastfeeding outcomes [9,10]. However, a meta-analysis of randomized controlled trials found that mobile app usage was not significantly associated with prolonged breastfeeding duration. However, only a few studies were included in the meta-analysis, and there was high heterogeneity among the interventions [11]. Recently published randomized controlled trials showed that the use of mobile apps can significantly increase [12] exclusive breastfeeding duration [13].

The Baby-Friendly Hospital Initiative (BFHI) was launched by the WHO and the United Nations International Children’s Emergency Fund in 1991 [14]. A large body of evidence shows that the BFHI can significantly improve breastfeeding outcomes [15,16]. The BFHI comprises 10 steps that aim to promote, protect, and support breastfeeding in a hospital setting [14]. Step 10 of the BFHI is about fostering the establishment of breastfeeding support groups in the community and referring breastfeeding individuals to them on discharge from the hospital or clinic [14]. Community support is essential for extending breastfeeding duration [17]. The Baby-Friendly Community Initiative (BFCI) is an extension of the BFHI [18] and is focused on providing community-based support for breastfeeding individuals [19]. However, the BFCI is implemented in only a few countries and resources from the government are needed to support the BFCI [19].

Globally, breastfeeding in public is not yet normalized. In Western countries, the proportion of individuals supporting breastfeeding in public ranges from 50% to 75% [20-23]. In China, although 65% of the community agrees that breastfeeding in public is acceptable, 30% believe that women should breastfeed only in private spaces [24]. Furthermore, breastfeeding individuals face numerous challenges when breastfeeding in public. Embarrassment has been identified as a key barrier to public breastfeeding [25] and unwanted attention and unsuitable structural environments are common challenges faced outside of the home [26]. Studies show that some public areas are unwelcoming to breastfeeding individuals [27], highlighting the lack of comfortable breastfeeding spaces [28]. A study has shown that breastfeeding in public is associated with a longer duration of breastfeeding [29]. It is crucial for breastfeeding individuals to find breastfeeding-friendly places when they visit public venues. Several mobile apps are available for breastfeeding individuals to locate breastfeeding facilities in their communities, such as “FeedFinder (GarbTech),” primarily used in the United Kingdom [30] and “Moommae (Moon Active)” in Thailand [31]. These apps can increase the confidence of breastfeeding individuals to go out and breastfeed [30] while supporting breastfeeding on demand [32]. However, users have emphasized the need for more breastfeeding facilities to be included in the app [30,32]. Therefore, we developed a social media mobile app with comprehensive territory-wide information on breastfeeding facilities in Hong Kong to promote breastfeeding-friendly attitudes within the community.

### Breastfeeding Practices and Public Breastfeeding in Hong Kong

In Hong Kong, approximately 84.7% of postpartum individuals initiate breastfeeding [33]. However, only 23.9% of parents are able to continue to breastfeed for 12 months and only 22.2% exclusively breastfeed until 6 months [34]. Breastfeeding individuals face various barriers in public places. Frequently reported unpleasant experiences included being stared at by others and being asked to breastfeed in unsanitary or unsuitable places, such as public restrooms [35]. A significant number of women stated that they reduced going out with their children and resorted to feeding them with infant formula to avoid public breastfeeding [35]. However, given that supplementing with infant formula is strongly associated with breastfeeding cessation [36,37], this is not an optimal solution. One study demonstrated breastfeeding in public was associated with longer breastfeeding duration [29]. Therefore, it is crucial to offer community support.

The objective of this paper was to describe the development of a breastfeeding-friendly app called “bfGPS (HKU TALIC),” which provides comprehensive territory-wide information on breastfeeding facilities in Hong Kong, with the goal of fostering a breastfeeding-friendly community. We also gain insights into how breastfeeding individuals and public venue staff perceive bfGPS through qualitative interviews.

## Methods

### Design

This study was conducted as part of the BFCI, which aims to promote breastfeeding-friendly attitudes in the community. The BFCI program encompasses the following activities: (1) providing training to the staff and management of public venues to support breastfeeding individuals, (2) offering breastfeeding support to pregnant and breastfeeding individuals, and (3) developing the bfGPS mobile app that aims to assist breastfeeding individuals in locating breastfeeding facilities in public venues in Hong Kong. The details of the BFCI program were described elsewhere [38].

**Figure 1.**
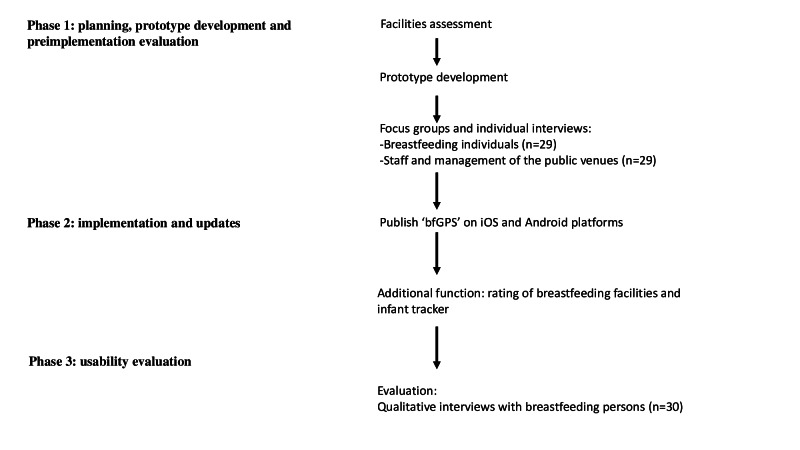
Flow diagram of the development of bfGPS.

In this study, we report the development of bfGPS, which is divided into three phases: (1) planning, prototype development, and preimplementation evaluation; (2) implementation and updates; and (3) usability evaluation (Figure 1). Phase 1 involves facilities assessment, the development of the prototype of bfGPS, and a preimplementation evaluation. The preimplementation evaluation involves focus groups and individual interviews to collect feedback on the app. For phase 2 of the development, we published bfGPS on iOS (Apple Inc) and Android (Google) platforms, and we updated the apps to add additional functions, which are rating of breastfeeding facilities and infant tracker. Phase 3 is a usability evaluation. It includes qualitative interviews with breastfeeding individuals to understand their experiences of using bfGPS.

### Phase 1: Planning, Prototyping, and Preimplementation Evaluation

#### Setting and Relevant Context

A meeting was held with experts, including maternal and child health researchers, app developers, breastfeeding individuals, and health professionals, to discuss the focus and functionality of the breastfeeding app. Based on the results of the Department of Health (2016) survey [35], the team decided to design an app that could offer user-friendly guidance on breastfeeding to the general public.

**Figure 2.**
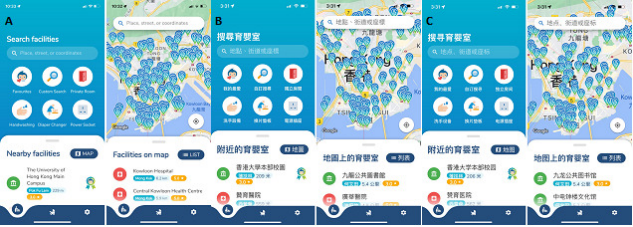
The interface of bfGPS for locating breastfeeding facilities is available in (**A**) English, (**B**) traditional Chinese, and (**C**) simplified Chinese. bfGPS provides a search bar that enables users to search breastfeeding facilities across Hong Kong. Users can view nearby facilities and the distance from their current location by enabling location access. The details of nearby facilities are shown with icons and descriptions. In addition, bfGPS allows for customized searches to enable users to filter results based on their needs, such as private rooms or handwashing facilities.

The content of the app was outlined, and a prototype of the bfGPS app was developed (Figure 2). bfGPS is available in English, Traditional Chinese, and Simplified Chinese. Between June 2019 and June 2020, undergraduate nursing students assessed breastfeeding facilities in various public venues in Hong Kong, such as government premises, shopping malls, and metro stations, using a structured checklist.

After developing the bfGPS prototype, breastfeeding individuals and staff from public venues were invited to participate in preimplementation evaluation. This preimplementation evaluation included focus groups and one-on-one interviews to gather feedback on the app’s functionality, design, performance, and suggestions for further development of bfGPS. Both public venue staff and breastfeeding individuals were recruited, as it was important to confirm the clarity of the content related to the public premises.

#### Recruitment and Data Collection

A total of 29 public facility staff members and 29 breastfeeding individuals were invited to participate in the focus groups and one-on-one interviews between May 2019 and October 2020. Convenience sampling was used to recruit participants. Public venue staff were recruited through social media advertisements or by participating in staff training within the BFCI program. Staff training for the BFCI program was described elsewhere [38]. In brief, a convenience sample of shopping malls and premises was identified throughout Hong Kong using the internet search. Invitation letters were sent to senior managers and customer service of the identified shopping malls and premises. The emails and letters of invitation include a description of the BFCI program, which included exposure to a breastfeeding mobile app, bfGPS. The shopping malls and premises employees were invited to attend a 2-hour training course based on the WHO breastfeeding counseling course. The employees who attended the BFCI training were invited to participate in the preimplementation evaluation through SMS text messages or emails.

The staff members had diverse occupations and job roles, ranging from frontline staff to managers. The inclusion criteria for staff members were individuals who were 18 years of age or older, worked in public venues, and interacted with the public as part of their jobs. Breastfeeding individuals were recruited if they had breastfed their infants in public or had used breastfeeding facilities in public venues. The recruitment was conducted through the researchers’ network. Participants received an HK $100 (approximately US $12.80) gift card as compensation.

### Phase 2: Implementation and Updates (Setting and Relevant Context)

Beta testing was conducted to provide early versions of the app to experienced app testers who examined it for errors, crashes, layout issues, software bugs, or other problems. The research team and app developers performed beta testing. bfGPS was published on iOS and Android platforms in September 2020. An app update was launched in September 2021 which allowed users to rate and evaluate breastfeeding facilities and included an online function for the public to upload recent information about breastfeeding facilities.

**Figure 3.**
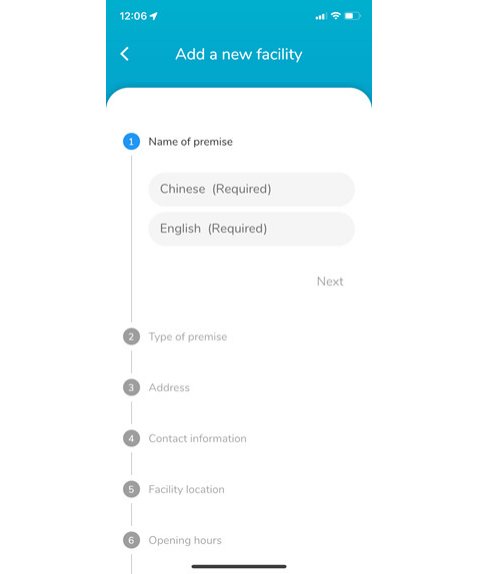
Interactive function for the users to upload information related to breastfeeding facilities in bfGPS. Users can submit information about breastfeeding facilities, such as name, type of premise, address, and photos of the premises. The study team then reviews the information to ensure accuracy before updating the app. This approach helps maintain up-to-date and reliable data for bfGPS users.

**Figure 4.**
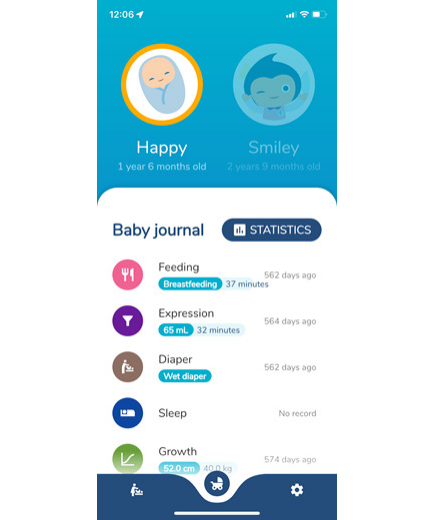
The infant tracker function of bfGPS. The infant tracker enables users to create multiple infant profiles. Key functions include recording infant feeding, human milk expression, diaper changes, sleep patterns, growth metrics, and immunization schedules. The growth patterns are plotted on growth charts using data from the Hong Kong Growth Study 2020 [39]. In addition, the infant tracker calculates upcoming vaccination dates based on the infant’s birth date, following the schedule of the Hong Kong Childhood Immunization Programme [40]. The infant record can be exported in a PDF file.

Based on the participants’ suggestions in the focus groups and one-on-one interviews, the ratings and evaluation stage were introduced in September 2021, along with an online function for the public to upload recent information about breastfeeding facilities (Figure 3). The infant tracker was a new add-on feature launched in May 2022 to encourage users to record infant development, including the amount of breast milk, breastfeeding times, sleep patterns, and reminders for feeding and vaccinations (Figure 4).

### Phase 3: Usability Evaluation (Setting and Relevant Context, Recruitment, and Data Collection)

Following the introduction of the infant tracker function, qualitative interviews were conducted with 30 breastfeeding individuals who used location mapping and the infant tracker function from August to September 2022. Breastfeeding individuals were recruited through Facebook (Meta) and Instagram (Meta) posts containing brief descriptions of the study and a link for study registration. The following criteria were used: (1) 18 years of age or older, (2) Cantonese speaking, (3) pregnant or recently gave birth to a live newborn, (4) Hong Kong residents, and (5) had ever used bfGPS. In the study registration form, participants were asked to indicate which functions did they used in bfGPS. Data saturation was achieved, as no new information emerged from the interviews [41]. Participants received HK $100 (approximately US $12.80) in coupons for a local grocery store as an honorarium for participating in the interviews.

### Data Collection

Semistructured interview guides with open-ended questions were used to collect the data. The participants’ experiences of using bfGPS, their insights into the strengths and limitations of bfGPS, recommendations for future development, and significant features for improving the user experience were comprehensively examined. The interview guides were refined as new insights, and perspectives were obtained throughout the study. Focus groups and one-on-one interviews were scheduled based on participants’ preferences and conducted at various locations. The interviews were conducted by the authors, who are experienced in conducting qualitative interviews (HSLF and KWL). For the preimplementation evaluation (phase 1), 7 focus groups and 1 staff one-on-one interview were conducted in public commercial rooms and at the university campus. The 3 focus groups and 18 one-on-one interviews with breastfeeding individuals took place in public commercial rooms. The usability evaluation interviews (phase 3) were conducted at public venues convenient for the interviewees (n=30). Each focus group and the one-on-one interview lasted approximately 30 minutes and were audio-recorded with the participants’ written permission.

### Data Analysis

Descriptive statistics were used to summarize the participants’ characteristics. Trained transcriptionists transcribed the audio recordings verbatim into Chinese. The transcripts were reviewed multiple times to increase the researcher’s familiarity with the participants’ experiences of using bfGPS. The transcripts were then denaturalized and translated into English. Content analysis was used to analyze the transcripts, as it allows for the identification of core consistencies and meanings in qualitative data [42]. The research team reviewed each transcribed interview and developed an open code list derived directly from the data. All textual data were coded and grouped into conceptually similar and overarching themes [43]. To enhance familiarity with the data, a manual data management approach was used, aided by using Microsoft Word [44].

### Ethical Considerations

Ethical approval was obtained from the Institutional Review Board of the University of Hong Kong and Hospital Authority Hong Kong West Cluster (UW18-489). The study was conducted in accordance with the principles of the Helsinki Declaration. Participants were informed that they could withdraw from the study at any time without any repercussions. They received HK $100 (approximately US $12.80) in grocery store coupons as an honorarium for interview participation. Written informed consent was obtained from all participants. No specific identifiers were included in the data used for analysis, and participants’ confidentiality was maintained at all times.

## Results

### Breastfeeding Facilities Assessment and Function of bfGPS

A total of 658 breastfeeding facilities in 502 sites were assessed, and the prototype of bfGPS was developed. bfGPS uses the GPS function to locate the nearest breastfeeding facilities. Users can use the advanced search function to narrow their search based on the type of premises and specific equipment provided by the breastfeeding facilities. In addition, photographs of the listed breastfeeding facilities were made available, along with equipment details.

### Preimplementation Evaluation of bfGPS

Tables1  2 present the characteristics of the staff members and breastfeeding individuals who participated in the focus groups and one-on-one interviews. Approximately 40% of the staff members were 35 years of age or above (Table 1). The majority of them were female (24/29, 83%), worked full-time (28/29, 97%), and were employed in the service industry (17/29, 59%). Table 2 provides the characteristics of the breastfeeding individuals, revealing that the majority were aged 35 years or older (15/27, 56%), primiparous (16/27, 59%), and returned to work within 6 months post partum (14/27, 52%).

**Table 1.** Characteristics of the public facility staff who participated in the preimplementation evaluation.

Demographic variable		Total (N=29), n (%)
**Age (years)**		
	18‐29	10 (37)
	30‐34	6 (22)
	≥35	11 (41)
	Not reported	2 (7)
**Sex**		
	Female	24 (83)
	Male	5 (17)
**Employment status**		
	Work full-time	28 (97)
	Work part-time	1 (4)
**Occupation**		
	Service industry	17 (59)
	Catering industry	4 (14)
	Property management	6 (21)
	Others	2 (7)

**Table 2.** Characteristics of the breastfeeding individuals who participated in the preimplementation evaluation.

Demographic variable		Total (N=27^a^), n (%)
**Maternal age (years)**		
	18‐29	2 (7)
	30‐34	10 (37)
	≥35	15 (56)
**Parity**		
	Primiparous	16 (59)
	Multiparous	11 (41)
**Age of the child during the interview**		
	<3 months	5 (19)
	3 to <6 months	5 (19)
	6 to <12 months	4 (15)
	≥12 months	13 (48)
**Return to work within 6 months post partum**		
	No	13 (48)
	Yes	14 (52)

a Of total, 2 participants had missing sociodemographic characteristics.

Participants provided feedback on various aspects of bfGPS, including layout, app performance, functionality, and suggestions for further development. Overall, the participants expressed satisfaction with the functions of bfGPS but preferred a more visually appealing layout. They also provided suggestions to enhance user experiences, such as implementing a rating function for the breastfeeding facilities and an infant tracker.

### Layout and App Performance

Most participants stated that the layout and interface of bfGPS were user-friendly and easy to use. The app effectively helped users locate breastfeeding facilities. While the participants found the facility photos useful, some expressed concerns about the loading speed and the app’s ability to quickly provide the information they needed, such as the location, name, and equipment details. Furthermore, participants recommended enhancing the visual appeal by improving the layout of bfGPS.

*With just one click to enter the page, you can choose locations and facilities, which are actually quite convenient. Moreover, opening hours were provided in the app. Parents might find it inconvenient to locate breastfeeding rooms, especially at night, which can be very helpful for them.*

[Staff #3, Focus group #1]

*When using the app for the first time, the premises’ name and address should load first…. Waiting for photos to load before showing the premises’ name and location can be time-consuming. We want to be fast.*

[Breastfeeding individual #7]

*bfGPS would attract me easily if it has an appealing appearance.*

[Breastfeeding individual #6]

### Areas of Improvement

Participants were asked to suggest ways to maximize the usage of bfGPS. In addition to the facilities locator function, participants recommended incorporating the following functions into the app: an infant tracker, more up-to-date information, increased uniqueness, and an interface for commenting on and reading other users’ feedback.

All participants expressed concerns about the accuracy and timeliness of the information provided by the app. They emphasized that having inaccurate or outdated information would cause inconvenience to them and discourage them from using the app. To address this, participants suggested adding a channel for app users to report updates on breastfeeding facilities. The system administrators can then verify and approve the updates, ensuring the app provides accurate and up-to-date information.

*I suggest the option for users to upload new baby-care facilities. Alternatively, companies can provide information directly to you (the research team) if they have opened new nursing rooms. You can check the information and upload it to the app after approval. If you need to do all the work by yourself, you may be unable to have a timely update…. This could significantly affect me if the information is outdated. If I go to the baby-care facility and discover it has been demolished, it will waste my time and mess up my plan*.

[Breastfeeding individual #8]

To prolong the usage of the app, participants suggested allowing them to enter feeding or baby information, such as feeding time or body weight of the infants. This feature would incentivize them to continue using the app and make them less likely to delete it.

*Honestly, my phone always runs out of storage space, making it easy to uninstall and reinstall bfGPS. However, if bfGPS provides more personalized functions, e.g. body weight record, feeding schedule, or which shopping mall I go to. I won’t delete the app. If I do not go out with the baby or only go to the shopping malls I am familiar with, I don’t see the need to keep the app as it uses up my storage space*.

[Breastfeeding individual #19]

*If the app includes a function for entering the baby’s height, weight, and head circumference, we are more likely to use it and keep it for an extended period*.

[Breastfeeding individual #1]

In addition to the standard information uploaded by the developers, participants expressed their desire to see the photos uploaded by other users and read their comments about the baby-care facilities.

*I would like to view photos taken by users*.

[Staff #4, Focus group #2]

Based on participants’ feedback from the preimplementation evaluation, we integrated an infant tracking function, incorporated public ratings, and added a function for uploading recent breastfeeding facility information in bfGPS. These enhancements were introduced in phase 2 of the study, as outlined in the methods section.

### Usability Evaluation

The characteristics of the participants are shown in Table 3. Approximately two-thirds were working full-time, and a majority had a bachelor’s degree or higher (17/30, 57%), were 30‐34 years of age (16/30, 53%), and lived in Hong Kong since birth (27/30, 90%).

**Table 3.** Characteristics of participants in the end-user evaluations.

Demographic variable		Total (N=30), n (%)
**Maternal age (years)**		
	18‐29	3 (10)
	30‐34	16 (53)
	≥35	11 (37)
**Employment status**		
	Full-time	20 (67)
	Part-time	2 (7)
	Stay-at-home parent	8 (27)
**Maternal education**		
	Lower than university degree	13 (43)
	University degree or above	17 (57)
**Length of residence in Hong Kong**		
	15 years or above	3 (10)
	Since birth	27 (90)

### bfGPS Improves Community Experience

The majority of participants reported that bfGPS is very convenient to use, providing prompt and detailed information about nearby breastfeeding facilities. bfGPS enables users to assess the breastfeeding facilities and plan ahead before going out with their babies. It offers flexibility to parents in their community experiences, as they can quickly locate the breastfeeding rooms.

*Before using bfGPS, I wouldn’t stay outside for too long. I would return home to feed within a few hours. With bfGPS, I know the locations of nursing rooms, allowing me to plan ahead for where I can go later. This enables me to explore farther places and extend the duration of my time going out*.

[Participant #14]

In addition, participants appreciated the rating function and the inclusion of photos of breastfeeding facilities, as it allowed them to gain insights into the settings of these facilities.

*The best feature of bfGPS is the provided photos, which clearly show the condition of the nursing rooms to me.… I think the ‘ratings’ function is beneficial because only those who have used the rooms would rate. The rating is important to me…I clicked on the page to see the issues of the breastfeeding facilities with 1-star ratings*.

[Participant #32]

### bfGPS Helps Track the Infants’ Growth

bfGPS allows people to track the growth of infants and helps them retrieve infants’ feeding records easily, especially during health checkups.

*bfGPS allows me to input my baby’s growth data, and it can generate a growth chart similar to the one in the maternal and child health center’s booklet. I find it beneficial, so I continue to record (the growth data) here…. The breastfeeding timer is also useful and convenient. It enables me to track how much milk or the duration that my baby was fed. I can observe the amount is increasing with the graph*.

[Participant #1]

*If my family member is willing to record in bfGPS, I can easily access information about my baby’s sleeping times. This allows me to plan her sleep schedule, especially on weekends, as I am the primary caregiver. My family member handwrites it, and I find it less convenient as I have to check the notebook each time*.

[Participant #18]

### Suggestions on Further Development

Similar to the findings in the preimplementation evaluation, the majority of participants emphasized the importance of providing written comments. In addition, participants highlighted the significance of sharing data from the infant tracker with family members. They suggested giving information on infant feeding and knowledge about infants’ growth and parenting into bfGPS.

*Users would benefit more from leaving comments after using the nursing rooms instead of solely relying on rating with the number of stars. For example, if the room is clean but the music is too loud, I can describe it clearly with words and leave other users to choose whether to use it. When you (the research team) receive the comments, you may communicate them to the person in charge and let them improve it*.

[Participant #19]

*It is important to have the sharing function, especially when other family members or helpers take care of your baby. This allows me to stay informed about how my baby is doing…. It is good to provide parenting information for parents. You may consider collaborating with the maternal and child health centers as they have a webpage on this*.

[Participant #9]

(*I would like to have information related to) the optimal time for introducing solid food or links to articles on the benefits of breastfeeding or other relevant breastfeeding.… It will be too monotonous if bfGPS is solely for record-keeping. It is better to have additional reference material. It will be challenging to continue using the app without information*.

[Participant #7]

### Evaluation of bfGPS

bfGPS was ranked number 3 in the Hong Kong App Store’s “Health and Fitness” free app category in September 2020. The total downloads in the iOS and Android platforms were 11,542 in December 2023. As of December 2023, the app usage data demonstrated impact and behavior change, with more than 11,000 unique users having used the app and performing more than 484,000 facilities searches. On average, each user conducted 50 (SD 7.25) searches, with an average of 4 (SD 2.47) searches per app usage session. All users viewed the facility details over 150,000 (SD 39,827.06) times in total. Each user viewed the detailed information of 19 (SD 3.43) facilities on average, and each session of app usage involved 1.5 (SD 0.77) views on average. The app attained average user ratings of 4.8 and 4.111 (out of 5.0) on the App Store and Google Play, respectively. We collected 2287 ratings of breastfeeding facilities and 217 uploads of breastfeeding facility information.

## Discussion

### Principal Results

This study provides an overview of the app development process of bfGPS, which primarily focuses on locating breastfeeding facilities in the community. We have refined the app based on the feedback provided by participants in the preimplementation evaluation, incorporating features such as ratings for breastfeeding facilities and the ability to upload recent breastfeeding facility information.

The infant tracker has the potential to increase the participants’ engagement. A study conducted in the United States showed that approximately 57% of participants used a mobile app to track infant feeding [45]. An infant tracker provides objective data to breastfeeding individuals regarding infant feeding methods and other aspects of baby care [46]. However, some breastfeeding individuals may express concerns about relying too heavily on the infant tracker feature [46]. Therefore, a large-scale quantitative study may be necessary to understand the effect of infant tracking on the well-being of breastfeeding individuals and its impact on breastfeeding outcomes.

For the future development of bfGPS and further research, we aim to transform it into a comprehensive platform that empowers users with access to breastfeeding knowledge and information, ultimately enhancing their self-efficacy and improving breastfeeding outcomes. We plan to collaborate with lactation consultants, registered nurses, midwives, breastfeeding individuals, and maternal and child health researchers to develop videos and informational leaflets. These resources will provide valuable information to help breastfeeding individuals overcome challenges. Furthermore, we intend to evaluate the effectiveness of bfGPS in supporting breastfeeding individuals within the community. The option to provide written comments on breastfeeding facilities is currently unavailable due to resource constraints, as close monitoring of the comments and additional staff is required. We will explore methods to enable a more comprehensive rating or comment system in the future.

The participants suggested the inclusion of breastfeeding information in the app. Studies have shown that incorporating breastfeeding information in the app can significantly improve breastfeeding outcomes [47,48]. While providing breastfeeding knowledge, it is essential to ensure the accuracy of the information. A study indicates that a high proportion of infant feeding apps provide incomplete or incorrect information [49]. In addition, most of these apps are provided by commercial sectors, which may result in biased information regarding infant feeding methods [49]. Beyond the app’s content, gamification can be an important element to incorporate, as it is associated with hedonic well-being [50] and can encourage long-term app usage [51]. By maintaining user engagement, gamification is an effective component for promoting behavior change [52].

The development of bfGPS involved cross-disciplinary and multidisciplinary collaboration among public, nongovernmental, governmental, and commercial organizations. The fundamental goal is to have an impact on health in Hong Kong and beyond, influencing public, nongovernmental, governmental, and commercial organizations, as well as policies to develop an infrastructure that transforms breastfeeding culture. To date, only a limited number of studies have been conducted to understand the effects of the BFCI on breastfeeding outcomes [19]. Further research is needed to understand the effective elements in creating a breastfeeding-friendly community.

The engagement of community partners and breastfeeding individuals during the app development phase is vital. This approach allows us to understand the community’s needs and ensures a user-centered design. Providing accurate information about breastfeeding facilities is important in encouraging breastfeeding individuals to use the app. Including commercial sectors in the BFCI program is important in gaining insights into the perspectives of the community members. This involvement enhances community engagement, encouraging community partners to offer breastfeeding-friendly services, and maintain up-to-date information on breastfeeding facilities. Ultimately, this collaborative effort contributes to fostering a breastfeeding-friendly attitude within the community.

### Limitations

Our study has several limitations. First, the end users who participated in the usability evaluation were recruited from the BFCI project’s social media page, which may have resulted in a selection bias, as participants were potentially more favorable toward breastfeeding, limiting the generalizability of the study findings. Second, conducting focus groups with public premises staff might have constrained them from freely expressing their individual opinions, as fellow participants were their colleagues or supervisors. Third, the objective of the usability evaluation was to understand the experiences of using the GPS feature. However, some participants did not use either the breastfeeding facilities locations or infant tracker functions, limiting their ability to provide feedback on these specific features. In addition, some focus groups and one-on-one interviews were conducted before the COVID-19 pandemic, while others were conducted during the pandemic. Participants’ concerns, needs, and expectations regarding breastfeeding facilities may have changed. This may limit the transferability of the findings.

### Conclusion

This paper presents the user-centered process used in developing the bfGPS app, which aims to improve the breastfeeding experiences of families in the community. In addition, the app includes an infant tracker feature as an add-on, which can enhance various aspects of infant care. Our objective is for this work to serve as a model or guide for applying a user-centered methodology when developing new health care apps. Ensuring the app provides accurate and up-to-date information on breastfeeding facilities is crucial to promoting its continued and widespread use among breastfeeding individuals. This approach holds promise as an effective method for enhancing breastfeeding experiences in public and the community. The app has the potential to both reinforce and support the breastfeeding information provided to parents while also offering strategies for self-management to users. The next step involves testing the app as a therapeutic tool through a randomized clinical trial to verify its effectiveness in sustaining breastfeeding duration and addressing immediate breastfeeding issues. This ultimate goal serves as the motivation behind the creation and development of the app.

## Abbreviations

BFCI: Baby-Friendly Community Initiative
BFHI: Baby-Friendly Hospital Initiative
WHO: World Health Organization
